# Editorial: Insights in consciousness research 2021

**DOI:** 10.3389/fpsyg.2023.1182690

**Published:** 2023-04-20

**Authors:** Narayanan Srinivasan, Luca Simione, Xerxes D. Arsiwalla, Johannes Kleiner, Antonino Raffone

**Affiliations:** ^1^Department of Cognitive Science, Indian Institute of Technology Kanpur, Kanpur, India; ^2^Institute of Cognitive Sciences and Technologies, Consiglio Nazionale delle Ricerche, Rome, Italy; ^3^Faculty of Interpreting and Translation, UNINT, Università degli Studi Internazionali, Rome, Italy; ^4^Department of Information and Communication Technologies, Pompeu Fabra University, Barcelona, Spain; ^5^Munich Center for Mathematical Philosophy, Ludwig Maximilian University of Munich, Munich, Germany; ^6^Munich Graduate School of Systemic Neurosciences, Ludwig Maximilian University of Munich, Munich, Germany; ^7^Association for Mathematical Consciousness Science, Munich, Germany; ^8^Department of Psychology, Sapienza University of Rome, Rome, Italy

**Keywords:** consciousness, phenomenal consciousness, complexity, non-local, hypnosis, meditation, EEG, microstates

In recent years, multiple theories have been proposed and are being tested to advance consciousness science (Seth and Bayne, 2022). Critical reviews focusing on these theories have proposed criteria for evaluating such theories (Doerig et al., 2021) and evaluated their potential convergence (Northoff and Lamme, 2021). The Research Topic on insights in consciousness research focuses on some critical aspects in consciousness research.

The paper by Marchetti focuses on the phenomenal aspects of consciousness (PAC), more specifically the “why” of the PAC, deriving an explanation for the evolutionary and functional understanding of consciousness. For him, PAC allows an agent to have a sense of self and provides information on how various mental operations influence the self. Marchetti uses a notion of information that is available only for an agent or self to understand the PAC, which forms the basis of conscious information processing. He argues that conscious information processing is due to two important components, self, and attention. In a functional perspective, self as a process reduces the complexity of the organism into a “single voice,” while attention focuses on specific aspects of the self. He argues that attentional activity on the state of self modulates the “energy level” of the neural substrate underlying the attentional activity. According to this perspective, different dimensions of PAC like quantitative, qualitative, hedonic, temporal, and spatial are associated with different features of modulation of energy level of the organ of attention and sense of self involved.

Amongst the prominent current theories of consciousness is the integrated information theory (IIT: Oizumi et al., 2014), which depends on measures of complexity (Arsiwalla and Verschure, 2018). There have been multiple criticisms of IIT [see Singhal et al. (2022) for a criticism based on temporal phenomenology]. The paper by Koculak and Wierzchoń argues that the focus of those studying the theoretical and empirical basis of IIT has been primarily on the states of consciousness and not directly on the contents of consciousness itself. They argue for the need to pay attention to complexity measures in understanding our conscious experience in terms of both states and contents of our conscious experience, while IIT provides only a quantitative measure of the degree of integration. The authors point to the need to dissociate the use of complexity measures from the ontological assumptions of IIT so that empirical studies on neural correlates of consciousness can study whether complexity measures can directly quantify properties of the contents of consciousness. One example they point out is the fMRI study by Boly et al. (2015) that measures Lempel-Ziv complexity under different stimulus conditions and found a difference in complexity. They argue that more studies investigating complexity measures under different conditions of consciousness (for example, conscious vs. unconscious perception) are needed.

Some have argued that we need to go beyond physicalist theories of consciousness. The paper by Wahbeh et al. briefly discusses physicalist theories including global workspace, higher order thought theories, IIT, and predictive processing/reentrant theories. They point to a lack of consensus regarding these theories, as these theories make different assumptions and explain address different phenomena. Then, the authors propose non-local theories of consciousness as a way to resolve the “hard problem” (Chalmers, 1996). They discuss a set of theories with many of them being of panpsychist nature, but it is also not clear that there is any consensus regarding these theories. Finally, they discuss a set of unexplained non-local phenomena which support non-local theories of consciousness. Authors then propose to consider in future research both local and non-local theories of consciousness and suggest how they could be further integrated.

In terms of states of consciousness, it is important to understand the phenomenological aspects and mechanistic underpinnings of different altered states of consciousness that include dreaming, hypnosis, and meditation. In this direction, the article by Penazzi and De Pisapia compares research and findings on hypnosis and meditation. The phenomenology of hypnosis includes dissociation, absorption, and suggestibility, while for meditation three general categories are described, i.e., focused-attention, open monitoring, and deconstructive meditation, which differ in terms of attention, metacognition, and experience of the self. Both hypnosis and meditation seem to involve relaxation, as they decrease the sympathetic response and increase the parasympathetic tone but differ in terms of volition and control (Dienes et al., 2022), as hypnosis is supported by an external subject with suggestive methodologies, while meditative states are typically self-induced. This leads to potential differences in meta-awareness between these two states with lesser meta-awareness in hypnosis and more meta-awareness in meditators, which can be understood in the context of HOT theory (Dienes et al., 2022). Finally, the authors point out the need for further studies comparing these conscious states, in particular, through EEG studies.

In fact, EEG is an optimal methodology to investigate altered states of consciousness, as it has a good temporal resolution, which allows to study transient and dynamics states of consciousness such as the so-called microstates, i.e., stable global patterns of electrophysiological brain activity that last about 100 ms. EEG recorded during different states of consciousness can be analyzed to identify such microstates. Bréchet and Michel discuss the presence of and changes in microstates during mind wandering, meditation, sleep and anesthesia. For example, they discuss the presence of two microstates during episodic memory retrieval, which is linked to fMRI resting state networks and the dynamics indicate switching between these microstates associated with different aspects of memory retrieval.

Since the operationalization of consciousness as a stream of information (James, 1890), we have since developed theories (Marchetti; Penazzi and De Pisapia; Wahbeh et al.), methods (Koculak and Wierzchoń), and technical skills (Bréchet and Michel) to advance our understanding of our experience as human beings. Overall, the papers included in this Research Topic give a glimpse of the complexity and open-endedness of the current debate in consciousness studies and contributes to consciousness research, which has a tremendous impact on research, clinical, and ethical aspects (Michel et al., 2019).

## Author contributions

All authors substantially contributed to this paper and approved it for publication.
